# Can treatment trial samples be representative?

**DOI:** 10.1016/j.brat.2009.06.019

**Published:** 2009-10

**Authors:** Jackie A. Wales, Robert L. Palmer, Christopher G. Fairburn

**Affiliations:** a University Department of Health Sciences, Brandon Unit, Leicester General Hospital, Leicester LE5 4PW, UK; b Department of Psychiatry, Oxford University, Warneford Hospital, Oxford OX3 7JX, UK

**Keywords:** Randomised controlled trials, Patient samples, Representativeness, Clinical utility, Eating disorders

## Abstract

A catchment area-based sample of patients recruited for an eating disorder treatment trial was compared with patients from the same geographical area seen in the 12 months before and after the trial. The three samples were very similar. The research sample was representative of the usual clinic sample from which it had been selected and thus the results could be extrapolated with some confidence to other similar clinical settings. It is concluded that whilst treatment trials, by their very nature, have explicit and implicit inclusion and exclusion criteria with appropriate designs they can be usefully representative.

## Introduction

Efficacy studies, especially randomised controlled trials (RCT), have been criticised; the most common objection being that they select patients with one diagnosis only and have a large number of exclusion criteria (Seligman, 1995), whereas in clinical practice many patients have multiple problems and co-morbid disorders. In 1997 Mitchell et al. (Mitchell, Maki, Adson, Ruskin, & Crow, 1997) hypothesized that the exclusion criteria employed in eating disorder treatment trials (e.g., high suicide risk, substance abuse) remove patients with a poor prognosis thereby inflating the apparent response rate. However Wilson (1998) in an appraisal of such trials concluded that they commonly included patients with levels of disturbance as severe as those seen in routine clinical settings.

This paper describes a study that was designed to assess the representativeness of patients treated for an eating disorder within a randomised controlled trial (RCT) by comparing the trial sample with patients seen in the same clinic before and after the period of recruitment for the trial. Between 2002 and 2007 the long-established Leicestershire Eating Disorder Service (LEDS) was one centre in a two-site randomised controlled trial comparing two forms of enhanced transdiagnostic cognitive behaviour therapy (CBT-E) (Fairburn et al., 2008, 2009). Patients were randomised to receiving one or other of these two therapies immediately or after an 8 week delay, a waiting list control condition. The two treatments were designed to be suitable for adult patients with any form of eating disorder of clinical severity provided only that they were considered suitable for outpatient treatment. Length of treatment was determined by the patient's body mass index (BMI) with those with a BMI over 17.5 receiving 20 weeks treatment and those with a BMI of 17.5 or below receiving 40 weeks.

The LEDS is the only specialised assessment and treatment service for adults with an eating disorder in the County of Leicestershire. It serves a total population of approximately one million. However, the catchment area for the trial was a sub-sample selected in advance by estimating the number of referrals needed to populate the trial. Patients are referred to the LEDS by family physicians and other clinicians. Thus patients referred to the service had been ‘pre-screened’ by another medical professional and deemed appropriate for a referral to a centre offering specialised eating disorder treatment.

The sample was intended to be “inclusive” with few exclusion criteria being applied (Fairburn et al., 2009). The selection criteria were designed to replicate the clinical filtering that takes place in routine clinical practice when selecting patients for outpatient treatment.

The specific aims of the present study were as follows:

1. To establish the referral rates of patients from the subsection of the LEDS catchment area in one year epochs before, during and after the trial;
2. To compare these three samples with respect to the proportions of patients who met the trial inclusion and exclusion criteria.

## Method

### Design

To assess the representativeness of the trial sample, the characteristics and disposition of the referrals to LEDs during a year when the trial was recruiting (2003) were compared with those of patients seen by the LEDS in the closest complete calendar years before and after the trial (2001 and 2006).

### Selection of participants for the trial

The participants in the trial were selected from consecutive referrals from a subsection of the catchment population of the LEDS. Recruitment commenced in March 2002 and continued until May 2005. The trial catchment area covered a population of approximately 571,000. The inclusion criteria were as follows:

■ Being a new referral or re-referral to the LEDS from within the trial catchment area.
■ Having an eating disorder requiring treatment, as judged both by the referring clinician and subsequently by the senior eating disorder specialist at LEDS (RLP).
■ Being between 18 and 60 years old.
■ Having a body mass index between 16.0 and 39.9.
■ Giving informed consent to participate in the trial.

The exclusion criteria may be divided into two categories, which may be termed “practical” and “clinical” respectively. It is the second group that is more relevant to the question of the extent to which a trial may be representative, or not, of usual practice. The practical exclusion criteria were as follows:

■ Not having a permanent address in the trial catchment area.
■ Not being sufficiently fluent and literate in English to complete interviews and questionnaires.
■ Being unwilling, or unable, to attend for 20 or 40 sessions of psychological treatment plus a possible additional waiting period of 8 weeks.

The clinical exclusion criteria were:

■ Being unwilling, or unable, to stop any other form of ongoing psychotherapy.
■ Being inappropriate to manage as an outpatient in the judgement of the local senior clinician (RLP); for example being at major risk of suicide.
■ Having a physical condition judged likely to complicate the interpretation of the trial's findings (e.g., a weight-losing illness, pregnancy). Other physical illnesses (e.g., diabetes mellitus) were not exclusion criteria.
■ Having a co-morbid axis I psychiatric disorder that required immediate active treatment. Those with stable propensity for instance a diagnosis of bipolar disorder were included but only if they were currently euthymic.
■ Having recently failed to respond to an evidence-based treatment for the same DSM-IV eating disorder, delivered by a specialist eating disorder service.

There was an initial screening of referrals by RLP. Those patients who were clearly ineligible as judged by the content of the referral letter (e.g., due to age, or location) were not assessed as part of the trial and were managed as usual by the LEDS. The remainder were seen for a first assessment interview to determine their eligibility. Those who were deemed eligible were then offered treatment within the context of the trial.

### Selection of the two comparison samples

Referrals from the same catchment area as the trial were identified. Two time periods were considered: the first complete years before the trial (January to December 2001) and immediately after the trial (January to December 2006). The same inclusion and exclusion criteria were applied, as far as was possible when examining patient notes retrospectively.

## Results

### Referral rates

Those patients who did not meet the age and BMI limits set for the trial were excluded from the analysis (2001 *n* = 16, 2003 *n* = 20 and 2006 *n* = 18) and are not considered further. All subsequent analyses relate to those who were potentially eligible.

As anticipated the referrals rates of such potentially eligible patients before, during and after the trial period were similar (see Table 1).

The proportion of patients eligible for treatment did not differ significantly over the three years (see Table 2). Similarly the percentage of those who, on consultation, did not have an eating disorder, were referred to other services or who were not offered therapy did not differ significantly in 2001 and 2006 from 2003. The main reasons for patients not being offered treatment were that they were moving away from the area or they did not return to complete their assessment.

**Fig. 1:**
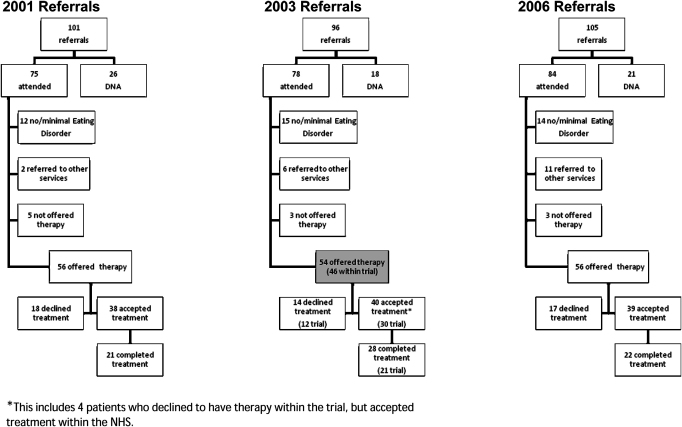
Flowchart of catchment area referrals received in 2001, 2003 and 2006.

Fig. 1 summarises these figures in the form of a flowchart.

In 2003, 8 patients (14.8%) (see shaded box in Fig. 1) from the catchment area were not offered therapy within the treatment trial and were treated within the LEDS. The main reason for this was that they could not commit to being available for the length of the trial due to their anticipating changing address (*n* = 4). The other reasons were: involvement with other services; weight having dropped below a BMI of 16 by the time of assessment, and illiteracy.

There were no significant differences in age, BMI, global EDEq scores and general psychiatric features, as measured by the BSI, between those offered therapy within the trial and the usual NHS provision (Table 3). It is, of course, possible that there are differences between these groups that do exist in unexamined characteristics which may have a bearing on treatment response. The main diagnosis for each of the groups was EDNOS, representing 60% (*n* = 27) of those treated within the trial and 87.5% (*n* = 7) of those treated within the NHS.

## Discussion

Can treatment trials be representative? The question may need to be broken in to two. Firstly, to what extent does the recruitment and pathway to the trial resemble the usual recruitment and pathway to treatment in the particular clinic or setting within which the research treatments are being offered? And secondly, to what degree does that clinic or setting reflect the universe of relevant clinics or settings? Those who are sceptical about the utility of trials may raise doubts in relation to either or both questions. Thus it is not uncommon for a trial to recruit by advertisement and screening, and then to offer free treatment within a special research unit but only to people with full syndrome disorders and no co-morbidity. In some circumstances research based care may be an affordable alternative for those without adequate insurance coverage. It is possible that such patients might have higher rates of medical or psychiatric co-morbid conditions that would make them less appropriate for care within a study protocol. Rates of transience and illiteracy might also affect take up and response. Nevertheless, research with samples recruited in this way may yield useful results. However, there can be only modest confidence about the extent to which the findings can be generalised to, say, the practice of a clinician working alone with patients who fund their own treatment and who have been referred regardless of “purity” of the diagnosis. Of course, it is the shared diagnosis that allows of any useful generalisation at all. It ensures that recruits to the trial and referrals to the clinic have similar core symptoms even if they differ in most other respects. Such lack of resemblance may perhaps not matter much for some disorders, such as cholecystitis, cataract or cervical cancer, but for most mental disorders, including eating disorders, such issues are likely to be important. Furthermore, reliance upon diagnosis alone as the basis of generalisation is more questionable where the relevant diagnostic systems are themselves seriously flawed, as is the case with the eating disorders (Fairburn & Bohn, 2005; Fairburn, Cooper, Bohn, O'Connor, Doll, & Palmer, 2007; Nielsen & Palmer, 2003). The example outlined above may not pass with confidence the test implied in either question. The method of recruitment, the terms of treatment, and the exclusion of many subjects because of the strict use of diagnostic criteria may not represent usual practice even within a specialised clinic and that clinic will not resemble at all closely the universe of clinics offering treatment for those with the relevant disorders.

So, treatment trials are often not representative in the relevant senses but can they be made to be? Or is there something inherent to treatment trials that makes them inevitably *un*representative. Some patients dislike the very idea of taking part in research. They do not want to be “guinea pigs”. Furthermore, the usually detailed consent procedures may be off-putting. All trials must have explicit inclusion criteria whereas this is not always the case for services or practices some of which use flexible and implicit criteria. And yet to avoid specified inclusion criteria would be to throw the baby out with the bath water. Much important progress in treatment research has been made because of the use of well-defined recruitment criteria.

The Leicester arm of the research was embedded in a well established specialist eating disorder service for adults which is a provision of the UK National Health Service for a defined catchment area. For those people referred to the service the trial treatments were offered to all potential patients subject to their meeting the broad criteria of the study and giving informed consent. Thus the trial treatments largely replaced the usual provision during the time of the trial. Recruitment of a sample for the trial that was closely representative of the usual clinical population was aided by the service (LEDS) being a near monopoly provider of the relevant type of care. Also the treatment offered in the trial did not differ much from the usual treatment offered in the service. Both the trial and usual practice offered time limited individual, face to face therapy. Had the trial involved a completely different type of treatment this might have affected take up and completion rates. Reassuringly with respect to the aspiration for the trial to be representative of the usual running of a clinical service, the cohorts of patients referred before, during and after the trial did not differ significantly in terms of their number, meeting the eligibility criteria for the trial, and their overall take up and completion of treatment. During the trial year studied (2003) of the 78 actually seen and assessed 28 completed treatment, of which 21 were in the trial. Such data presented in a CONSORT diagram might be thought of as evidence of the selectivity of research samples. However, it is important to note that comparison with the non-research cohorts presenting from the same catchment area in the years before and after the trial demonstrates that the proportion of people completing treatment was remarkably similar (Fig. 1).

We consider that this study demonstrates that treatment trials can be usefully representative although the degree to which they are depends upon the details of design. We recommend that reports of treatment trials should include consideration and discussion of the ways in which their samples resemble and differ from those usually seen in clinical practice.

## Figures and Tables

**Table 1:** Referral summary.

Year	2001 (before trial)	2003 (during trial)	2006 (after trial)	Significance
Total referrals	101	**96**	105	*χ*^2^ = 0.617, df = 2, *p* = .734
Did not attend	26 (25.7%)	**18** (**18.8%**)	21 (20.0%)	*χ*^2^ = 1.646, df = 2, *p* = .439

**Table 2:** Outcome of assessments for catchment area referrals 2001, 2003 and 2006.

Outcome of consultation	2001 *n* = 75	2003 *n* = 78	2006 *n* = 84	Significance
Eligible for treatment	56 (74.7%)	**54** (**69.2%**)	56 (66.7%)	*χ*^2^ = 1.245, df = 2, *p* = .537
No eating disorder	12 (16.0%)	**15** (**19.2%**)	14 (16.7%)	*χ*^2^ = 0.364, df = 2, *p* = .834
Referred to other services	2 (2.7%)	**6** (**7.6%**)	11 (13.1%)	*χ*^2^ = 5.860, df = 2, *p* = .053
Not offered treatment	5 (6.7%)	**3** (**3.8%**)	3 (3.6%)	*χ*^2^ = 0.629, df = 2, *p* = .794^a^

a Exact Pearson's chi-square used.

**Table 3:** Clinical characteristics of patients offered therapy.

	Offered trial therapy, *N* = 46, mean (SD)	Offered NHS therapy, *N* = 8, mean (SD)	Significance
Age	25.5 (6.8)	27.6 (5.2)	*t* = .837, df = 51, *p* = .406
BMI	22.5 (4.2)	23.2 (7.2)	*t* = .408, df = 51, *p* = .685
Global EDEq^a^	4.28 (1.12)	3.52 (1.49)	*z* = 1.3, *p* = .207
BSI^b^	1.94 (0.8)	2.34 (0.8)	*t* = 1.007, df = 43, *p* = .320

a Eating Disorder Examination Questionnaire (Fairburn & Beglin, 1994).

b Brief Symptom Inventory (Derogatis & Spencer, 1982).
